# The potentially neglected culprit of DC surface flashover: electron migration under temperature gradients

**DOI:** 10.1038/s41598-017-03657-1

**Published:** 2017-06-12

**Authors:** Chuanyang Li, Jun Hu, Chuanjie Lin, Jinliang He

**Affiliations:** State Key Lab of Power Systems, Department of Electrical Engineering, Tsinghua University, Beijing, 100084 China

## Abstract

This report intends to reveal the role of electron migration and its effects in triggering direct current (DC) surface flashover under temperature gradient conditions when using epoxy-based insulating composites. The surface potential and the surface flashover voltage are both measured using insulators that are bridged between two thermo-regulated electrodes. The space charge injection and migration properties under different temperature are detected. The results show that the surface potential rises significantly because of electron migration near the high voltage (HV) electrode under high temperature conditions, thus creating an “analogous ineffective region”. The expansion of this “analogous ineffective region” results in most of the voltage drop occurring near the ground electrode, which serves as an important factor triggering positive streamers across the insulation surface. This work is helpful in understanding of DC surface flashover mechanism from a new perspective and also has important significance in design of a suitable DC insulator to avoid surface flashover problem.

## Introduction

Solid dielectrics are commonly used as electronic components in various fields, including aerospace, military, telecommunications, electronic and electrical (or power) engineering applications^1–4^. Any electrical devices that use gases or a vacuum as their electrical insulating medium must also contain some solid dielectrics at certain points, either to mechanically support or to separate the conductors^5, 6^. It is believed that bridged insulation always fails by flashover across the solid surface at a lower voltage than that required to cause failure of an unbridged gap of the same dimensions; this is due to the distortion of the electric fields in the triple junctions^7–13^. Considerable efforts have been made to investigate surface flashover under alternating current (AC) voltages^11–13^. However, stable operation of insulating materials under DC voltages has been a problem in various engineering fields, and has limited the development of the high-voltage electric power and pulsed power equipment. For example, the surface discharge takes place on the insulation surfaces of spacecraft operating in high vacuum environments, and the surface flashover sometimes occurs for unknown reasons along the insulators that are used in DC power transmission and distribution units^14–18^.

**Figure 1:**
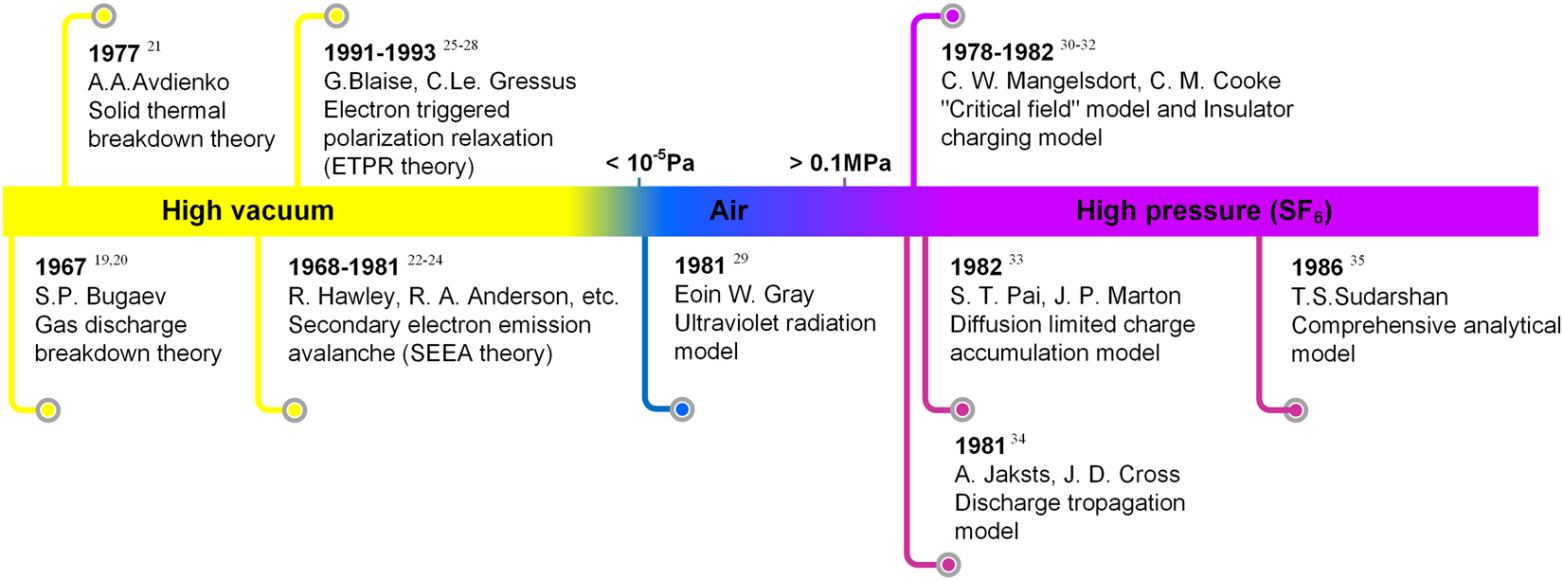
Generally accepted models of the surface flashover mechanism in vacuum and high pressure.

Figure 1 shows the generally accepted models of the surface flashover mechanism in vacuum and under high pressure^19–35^. It can be found that the surface flashover phenomenon under vacuum conditions have been explored since several decades ago. To data, two main models have been generally accepted to illustrate the surface flashover mechanism in vacuum: the secondary electron emission avalanche (SEEA) model and electron triggered polarization relaxation (EPTR) model. However, there is still disagreement concerning the mechanism and details in case of the development stage of the surface flashover^36, 37^, which may be largely because of the numerous factors that can affect the flashover characteristics, including the electrode-dielectric contacts, the angle between the electrodes and the dielectric surface, the dielectric material used, the temperature and vacuum levels of the ambient atmosphere, and electrical pre-stressing^38–41^. Additionally, some of the hidden factors such as charge injection or migration along dielectrics may influence the progress of the intermediate stage. For bridged insulation in high pressure, surface charge problems under AC and DC conditions have become more prominent since SF_6_ was used as an arc-extinguishing medium in the 1920s. Currently, although Prof. Sudarshan has put forward the “comprehensive analytic model” which could analyze the surface flashover mechanism under high pressure comprehensively, the charge migration due to injection of conductors, space charge migration and/or trapping on the surface may be a neglecting potential factor that affect the surface breakdown of current-carrying equipment^42–45^. It has been reported that the annual losses that arise from surface flashover on vacuum surface discharge or equipment such as high-voltage DC bushings, circuit breakers and gas insulated switchgear (GIS) spacers under DC voltage application are measured in millions of dallors^46^. In China, with the development of west-to-east electricity transmission projects and regional interconnected power network, the construction of ultra-high voltage interconnected grid becomes an imperative requirement^47^. Many failures of the electric power systems, especially in 1000 kV power systems, result from surface flashovers of spacers inside GIS, for unknown reasons^45, 48^. Therefore, it is of great practical significance to explore the hidden mechanism of the surface flashover phenomenon.

**Figure 2:**
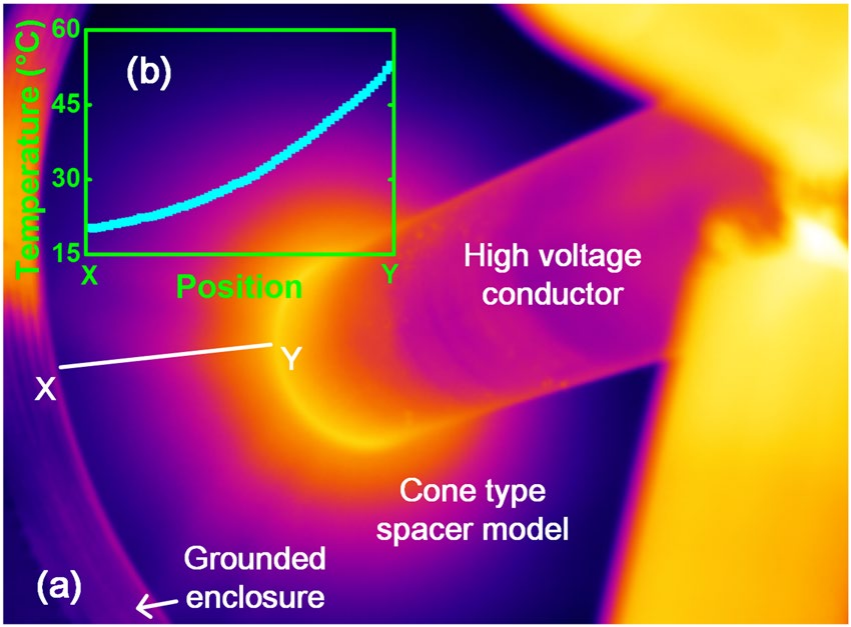
(**a**) Infrared imaging of cone type spacer model with the temperature of the high voltage conductor reaching 60 °C after 20 min in ambient environment and (**b**) temperature gradient from the grounded enclosure (X) to the high voltage conductor (Y).

During the operation of DC energy systems, the conductor temperature fluctuates because of Joule heating caused by the changing conducting current or by long-term operation, and can sometimes reach up to 80 °C–90 °C^49–51^. This means that, in the case where energy transmission occurs, the temperature of the conductor could be in the range from ambient temperature to very high temperatures in some regions while operating under heavy load conditions^52, 53^. Figure 2 shows an infrared image of a cone-type spacer model, where the temperature of the high voltage conductor reaches 60 °C after 20 min in an ambient environment. It can be seen from Fig. 2(b) that a temperature gradient has been created across the insulator when the current was flowing in the inner conductor. It is because that the thermal conductivity of the insulator (in range of 0.65–0.7 W·m^−1^·K^−1^ for epoxy and Al_2_O_3_ composite) is much larger than the surrounding atmosphere which is usually 0.01206 W·m^−1^·K^−1^ for SF_6_. Based on the above discussion, in any investigation of the relationship between charge migration and surface flashover under DC conditions, the temperature gradient should undoubtedly be taken into account.

In our recent research, we intended to clarify the effects of charge migration in an epoxy and alumina composite on the surface flashover phenomenon under temperature gradient conditions. The surface flashover inception voltage and the corona inception voltage were observed. Using a combination of a surface potential detection system and thermal imaging techniques, we detected the synchronization of the surface potential with the temperature distribution on the insulation surface. A large reduction in the surface flashover voltage has been found to occur when the temperature gradient is taken into account. To explain this unexpected phenomenon, an analogous ineffective region is proposed based on the shift in the surface potential. The experimental results presented here is helpful in understanding of DC surface flashover mechanism from a new perspective and also will notably influence future research directions and modification solutions for DC or high-voltage (HVDC) dielectric materials.

## Result and Discussion

### Surface potential distribution

**Figure 3:**
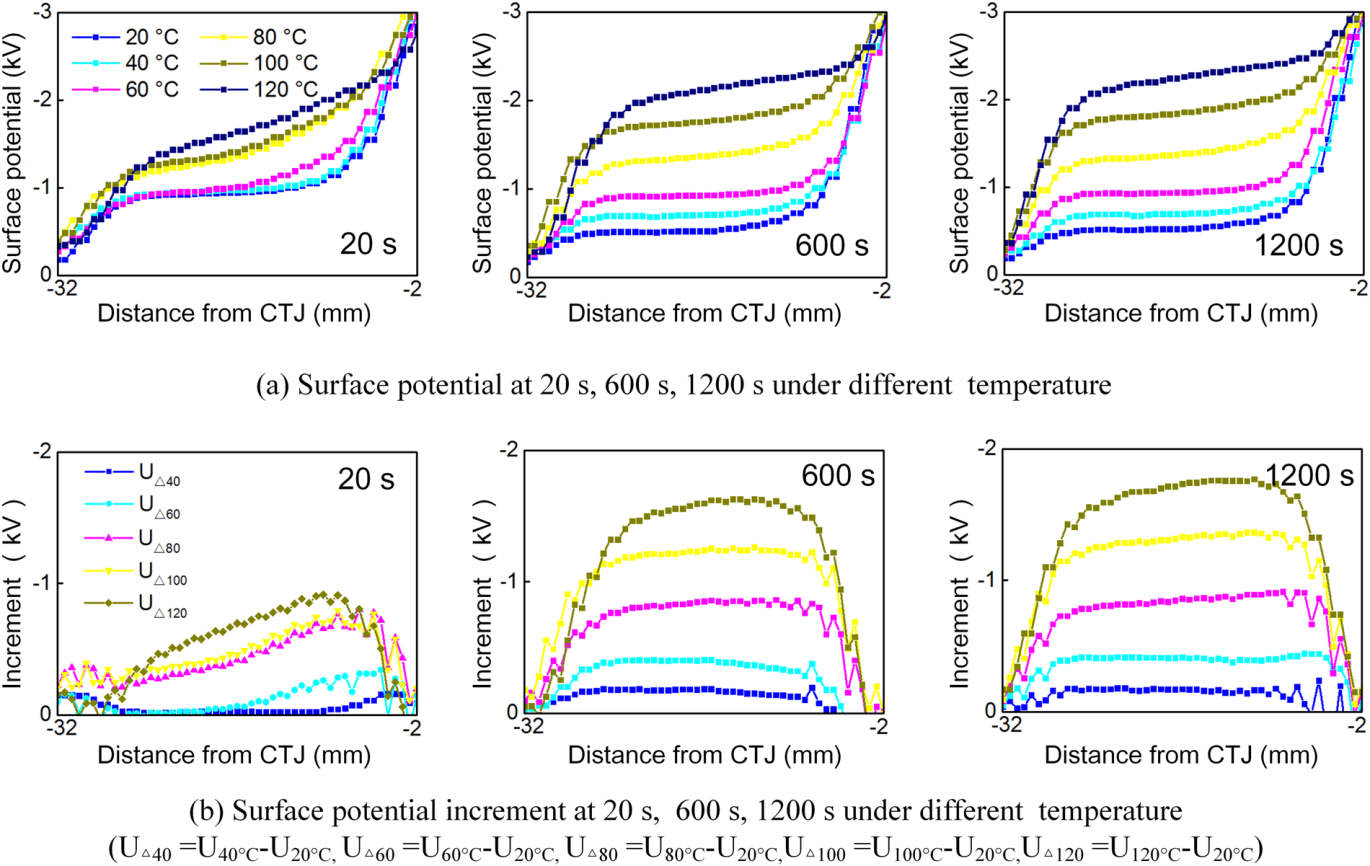
(**a**) Surface potential variation under different temperature gradient at different time duration and (**b**) surface potential increment under different temperature.

The results shown in Fig. 3 demonstrate the variation of the surface potential with respect to both time duration and heat propagation when using an epoxy-based alumina composite. It can be found in Fig. 3(a) that the surface potential rises with time at 20 s, 600 s, and 1200 s under the same temperature conditions. Simultaneously, when compared with the surface potential curve that was measured at 20 °C, as shown in Fig. 3(a), a significant increase in the surface potential is found near the HV electrode when the HV electrode temperature increases from 40 °C to 120 °C. Additionally, the increments in the surface potential in the different time periods also show an increasing trend with increasing temperature, and the growth rate of the potential when measured at 120 °C was nearly twenty times the rate that was measured at 20 °C after 1200 s, as shown in Fig. 3(b).

### Temperature propagation and streamer development

**Figure 4:**
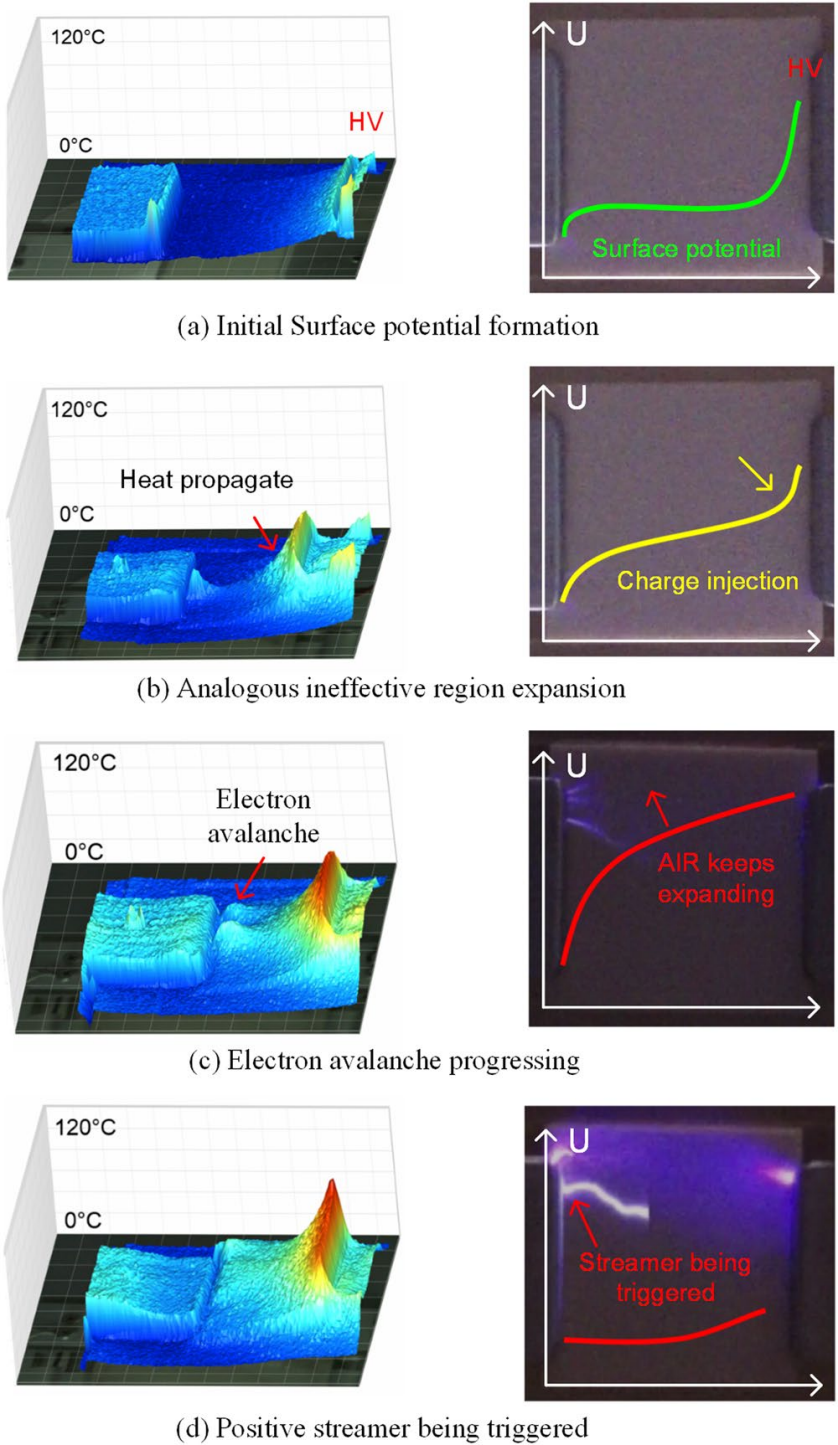
Thermal image and surface streamer picture with the temperature of the HV electrode increasing from 30 °C to 100 °C at 35 kV: (**a**) Initial potential formation stage, (**b**) electron avalanche incepted due to formation of “analogous ineffective region”, (**c**) electron avalanche progressing, and (**d**) surface flashover being triggered.

Figure 4 shows a thermal image and a surface streamer picture when the temperature of the HV electrode increased from 30 °C to 100 °C under application of a voltage of −35 kV. When the voltage was initially applied, charges were injected from the HV electrode and a slight increase in surface potential can be found near the HV electrode, as shown in Fig. 4(a). As the temperature increases, the free electrons in the impurity level that were excited by the thermal vibration interaction of the lattice migrate into the conduction band, and then shift through the bulk and the surface when driven by the electric field. These charges transfer across the surface barriers and take part in the energy conduction process, which causes reductions in both the volume resistivity and the surface resistivity with increasing insulation temperature, and results in high potential occurring near the HV electrode. Additionally, the thermal electron motion can accelerate the charge accumulation rate in the high conductivity surface area and this leads to a gradual increment in the potential in the hot regions of the dielectrics, as shown in Fig. 4(b).

Because of the heat propagation, the increased temperature in the insulation areas near the HV electrode leads to an increase in the number of hot electrons that migrate from the bulk to the surface, which then promotes the surface potential in the high temperature dielectric area. Consequently, the surface potential continues to rise until it is finally close to the conductor potential. The dielectric position at which the surface potential reaches more than 60% is defined as the analogous ineffective region, which causes most of the voltage drop to occur across the insulation area near the ground electrode. Therefore, the dense electric field is likely to cause gas ionization at the triple junction of the electrodes, thus forming the electron avalanches that are shown in Fig. 4(c). As the heat continues to propagate in the insulation, the analogous ineffective region continues to expand, and this results in a reduction of the effective insulation distance near the ground electrode. As a consequence, most of the voltage drop is applied to a small insulation region near the ground electrode, and this creates a very dense electric field. In addition, the electron thermal motion in the electron avalanches strengthens and hits the insulation surface at the triple junction of the ground electrode, which in turn increases the temperature at the insulation triple junction and promotes the development of the electronic avalanches shown in Fig. 4(c). Finally, even when the temperature has stabilized, the hot electron migration and accumulation in the surface causes the analogous ineffective region to expand continuously from the HV electrode to the ground electrode, and results in a greatly distorted electric field near the triple junction of the ground electrode. The electron avalanche in the strong electric field develops rapidly, and as a result, the electron density at the front of the electron avalanche becomes very high. Additionally, photon emission is generated by the recombination of electrons with the positive charges in the space. Part of the photon is absorbed by the positive space charges, and forms a secondary electron emission avalanche (SEEA). The front of the SEEA recombines with the positive space charge, and finally positive streamer propagation from the ground electrode to the HV electrode is developed as shown in Fig. 4(d).

The streamer finally neutralizes the surface charge and also brings increased heat to the surface. Because the power supply is not sufficient to sustain arc development, the streamer is extinguished when the arc has bridged the electrodes. Surface charge can then be accumulated and a new surface potential is rebuilt over a short period of time, thus triggering another positive streamer. Because the development of electron avalanches is random, streamer propagation across the surface can be an astatic process. In addition, it takes time to complete rebuilding of the analogous ineffective region, and therefore, a delay time is experienced before the surface flashover occurs under direct voltage application.

### Surface flashover and corona inception voltage

**Figure 5:**
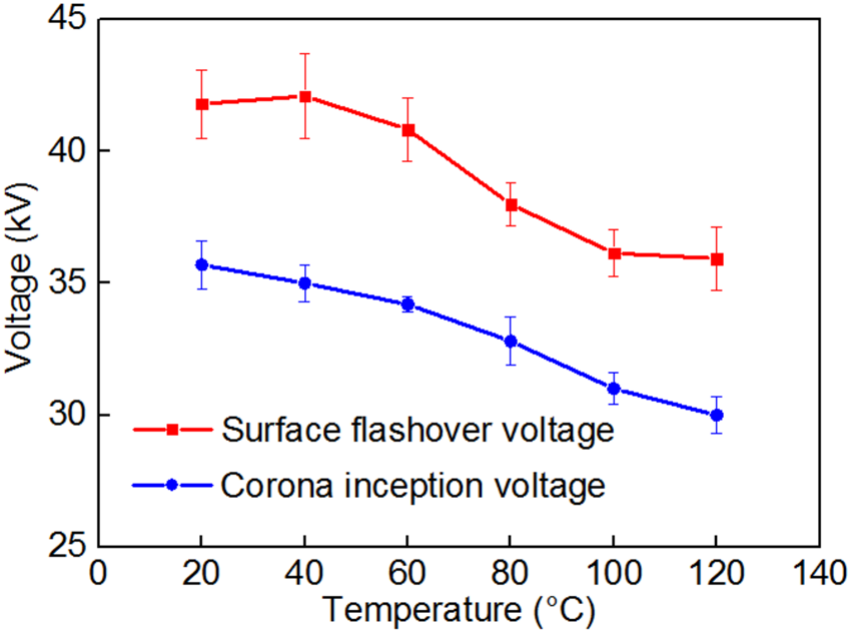
Surface flashover voltage and corona inception voltage at different temperature.

Figure 5 shows the surface flashover voltage and the corona inception voltage of the experimental sample under different temperature gradient conditions. When the applied voltage starts increasing, the electric field shows a dense concentration at the triple junction, where the corona initially occurs before surface flashover. In addition, the expansion of the analogous ineffective region caused by the temperature gradient results in descending trends in both the surface flashover voltage and the corona inception voltage. When the HV electrode temperature was 20 °C, the inception voltages of the surface corona and the surface flashover remained in the ranges up to 35.5 kV and 42 kV, respectively. When the temperature of the HV electrode increases, the inception voltages of the surface corona and the surface flashover continuously decrease. When the temperature of the HV electrode reached 120 °C, the inception voltages of the surface corona and the surface flashover fell below 30 kV and 32 kV, respectively.

### Mechanisms of the analogous ineffective region

According to energy band theory, charges in ideal dielectric materials are captured in the valence band under normal conditions, and only a small number of charges are located in the conduction band^54^. However, the impurities that are introduced in the manufacturing process inevitably bring high energy levels to the band gap. Charges that are trapped in the impurity energy levels are excited and reach the conduction band, where they form free conduction band charges. Based on the Schottky and Fowler-Nordheim effects, when operating under low temperature conditions, charges that are driven by the electric field are injected from the cathode into the conduction band of the dielectric material. Simultaneously, electrons are transferred through the surface states by the hopping mechanism when they obtain sufficient energy from external stimulation. Because of the high-density trap levels inside the dielectric material, the charges are trapped, and this trapping process gradually slows down and dynamic equilibrium is reached after a certain period of time^55^. Consequently, the surface charge transfer rate, which is determined by the electrode shape and its material properties, is reduced. We believe that by excluding external interference sources such as ultraviolet illumination or electroluminescence by charge injection at the cathode triple junction^56^, the charge migration in some cases is not necessarily harmful in triggering the surface flashover, unless heat transfer is encountered.

When a DC voltage is applied to the dielectric material, the electric field initially shows a capacitive distribution that is dependent on the permittivity of the dielectric material, and it then forms a stationary resistive distribution that is governed by the bulk resistivity and the surface resistivity of the insulating material^57^. When the temperature of the HV electrode increases, the heat is transferred very rapidly in the metal conductor because heat transmission in alumina is carried out on the basis of free electron collisions. However, the free electrons are bounded in the dielectric material, and heat propagation must be accomplished via the vibration of the lattice, which causes the heat conductivity to be lower than that of the alumina by more than ten times in some cases. Therefore, a stable high temperature region is formed in the HV electrode within a short period of time. Simultaneously, the heat propagates via forms of heat conduction across the interface to the dielectric material, and a temperature gradient is finally formed across the dielectrics. The foundations of this process usually take more time, and are dependent on the heat conduction rate of the dielectric material. As the heat propagates from the HV electrode to the dielectrics, the surface resistivity and the bulk resistivity in the area near the HV electrode both drop accordingly.

**Figure 6:**
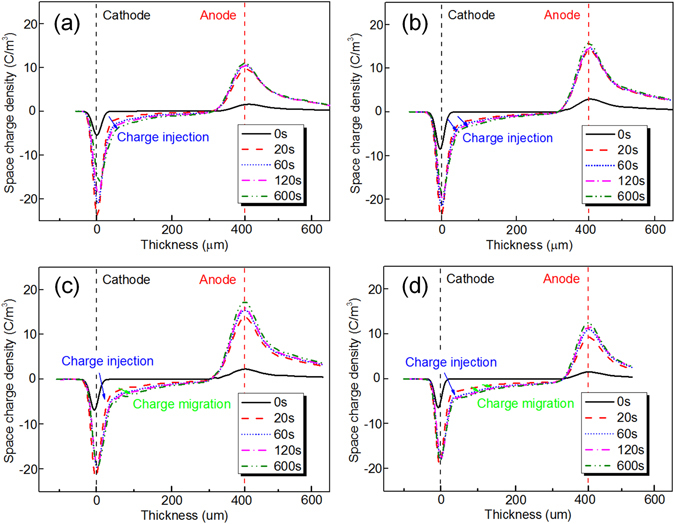
The space charge distribution of experimental samples subjected to a dc electric field of 40 kV·mm^−1^ for 600 s under different temperature: (**a**) under temperature of 30 °C, (**b**) under temperature of 60 °C, (**c**) under temperature of 80 °C, and (**d**) under temperature of 100 °C.

**Figure 7:**
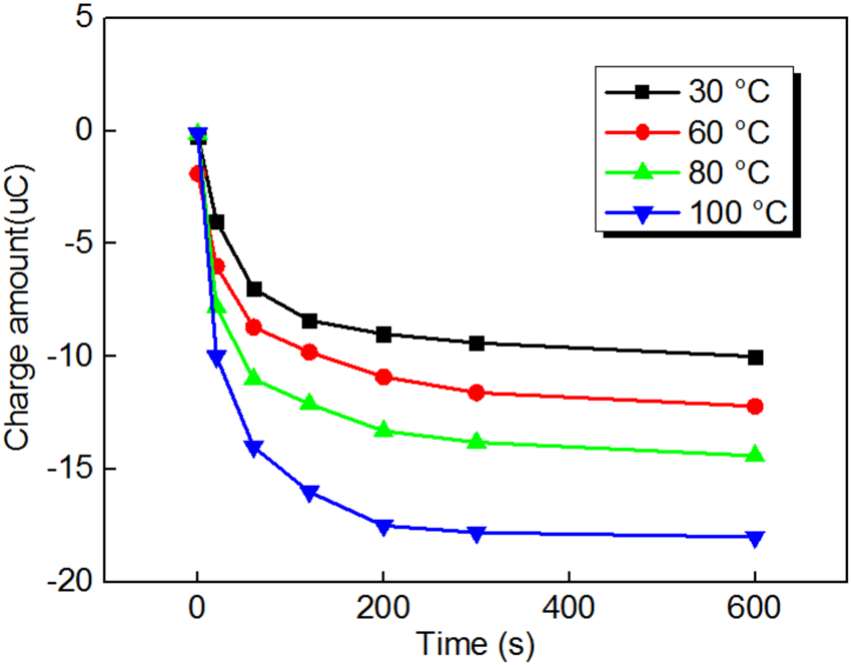
The space charge injection amount for experimental samples subjected to a dc electric field of 40 kV·mm^−1^.

For a more direct understanding of charge injection and migration phenomenon under different temperatures, space charge test is performed and the results are shown in Fig. 6. It is found that charges are injected continuously from the cathode to dielectrics under normal temperature from 0 s to 600 s shown in Fig. 6(a). With the rising of the temperature, the injected charge amount increases and injected charges start migrating from the cathode to the anode, which can be found in Fig. 6(b). When the temperature reaches 80 °C, the charge injection phenomenon becomes more obvious within the first 120 s and injected charges are more easily migrating from the cathode to the anode, shown in Fig. 6(c). When the temperature reaches 100 °C, the charge injection and migration behavior becomes much more obvious within the first 120 s. Afterwards, the injected charge amount reaches a saturate stage after 120 s and a state of equilibrium is reached, shown in Fig. 6(d). Also, we calculated the charge injection amount from the cathode and the result is shown in Fig. 7. It is found in Fig. 7 that the charge injection amount increases with the time increasing. The amount of charge injection increases from the original charge amount of less than 10 pC under 30 °C to more than 15 pC when the temperature increases up to 100 °C. This suggests that charge carriers are more easily injected from the cathode. Besides, the injected charges trapped near the cathode can form a dense electron region, which creates a reversed electric field that prevent charge injection from the cathode^43^. Under high temperature, the trapped charge near the cathode transports in dielectrics decreases the reversed electric field near the cathode, which is propitious to charge injection. In addition, as shown in Fig. 3(b), the surface potential increment curve shows basically saturated trend which again validates the dynamic equilibrium of charge injection and migration.

**Figure 8:**
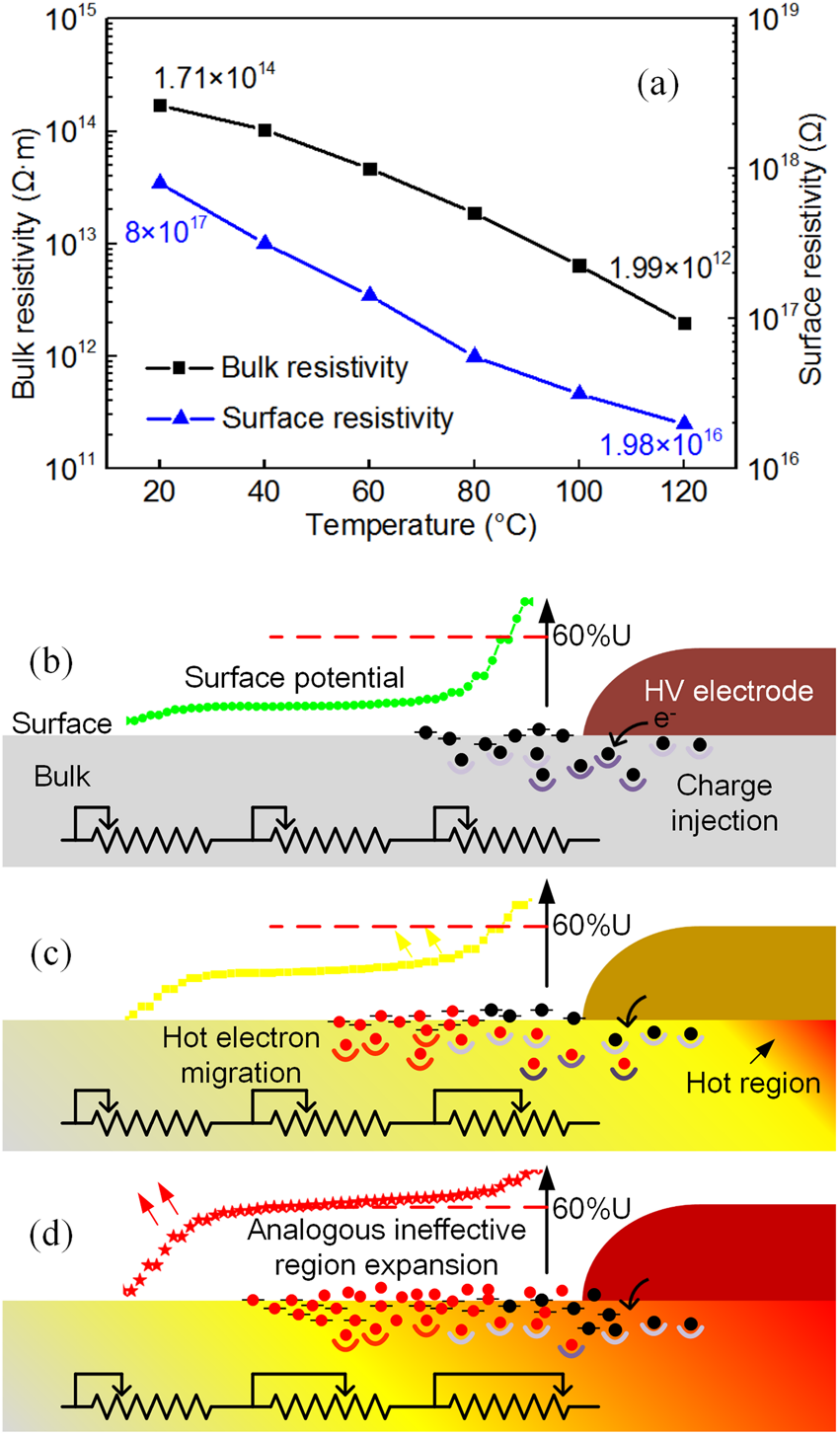
(**a**) Bulk resistivity and surface resistivity variation with temperature and schematic diagrams for surface potential increase with temperature: (**b**) Surface potential increase due to charge accumulation on the insulation surface. (**c**) Hot electron migration with the increase of temperature in the insulation near the HV electrode. (**d**) Expansion of “analogous ineffective region” due to hot electron migration.

Also, we tested the resistivity of the experimental sample and found that both the bulk resistivity and the surface resistivity decrease from their original values of 1.71 × 10^14^ Ω·m and 8 × 10^17^ Ω to 1.99 × 10^12^ Ω·m and 1.98 × 10^16^ Ω, respectively, when the temperature increases from 20 °C to 120 °C, as shown in Fig. 8(a). From a macro perspective, the bridged insulation can be construed to be a series of adjustable resistances that are regulated by the temperature. Under low temperature conditions, the dielectric remains in a good condition, as depicted in Fig. 8(b). In this process, dynamic equilibrium is reached and the surface potential can hardly be changed by any charge transfer activity. As the temperature increases in the insulation near the high voltage conductor, electrons that were trapped in the impurity energy levels are excited by the heat propagation and can then reach the conduction band, forming hot electrons^58^. The hot electrons in the conduction band are difficult to stabilize and can migrate through both the bulk and the surface. The hot electrons are finally trapped in the surface states and release their energy^59^. The gradual increase in the resistance value in this region also provides an explanation for the charge migration behavior. Therefore, an increase in the surface potential is found near the HV electrode at high temperatures. Additionally, it is suggested that during this process, the increased bulk and surface conductivities are favorable for electron migration through the insulation layer, which can then promote the injection of charges from the HV electrode. Consequently, large numbers of surface charges are injected and migrate through the insulation layer, and this can enhance the surface potential in the high temperature region, as shown in Fig. 8(c). As the temperature continues to rise, the resistance near the conductor decreases significantly because of the migration of the electrons, and the bulk resistivity in the dielectric region near the HV electrode is measured to be lower than the original value by nearly two orders of magnitude, as shown in Fig. 8(a). As a result, the surface potential in this region can increase up to more than 60% of the applied high dc voltage. This is defined as the analogous ineffective region, which causes most of the voltage drop to be near the ground electrode, as shown in Fig. 8(d).

**Figure 9:**
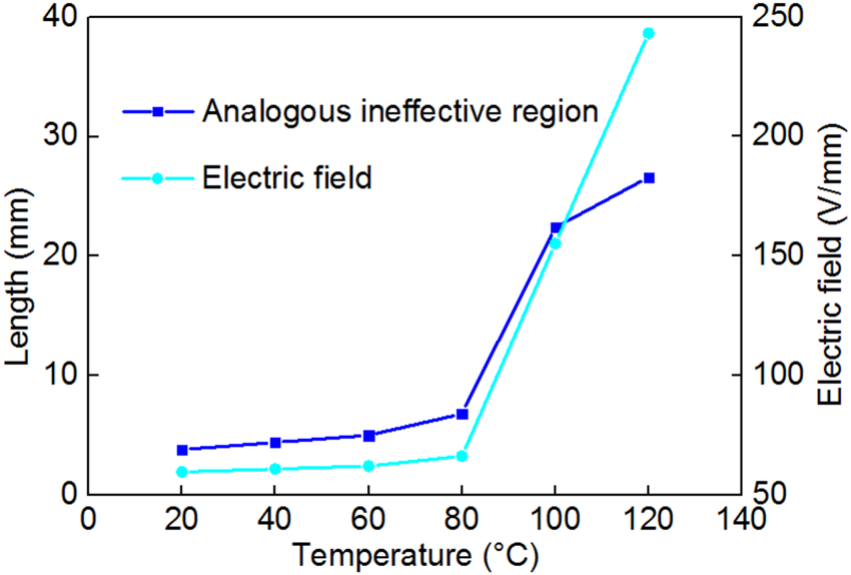
Length of “analogous ineffective region” and the electric field near the ground electrode at different temperature.

With the expansion of the analogous ineffective region, the electric field strength near the ground electrode becomes distorted with respect to the increase in temperature. The length of the analogous ineffective region and the electric field near the ground electrode at different temperatures are shown in Fig. 9. In our experiment, with expansion of the analogous ineffective region, which grows from less than 5 mm in length to more than 25 mm in length, the electric field increases significantly to more than five times its original value at 20 °C when the temperature reaches 120 °C. It is important to note that the assumption of a uniform electric field between the ground electrode and the edge of the analogous ineffective region can cause the maximum electric field at the triple junction of the ground electrode to be somewhat underestimated.

## Conclusion

We have investigated the surface potential and the surface flashover voltage under temperature gradients in combination with a thermal imaging technique. The results suggest that charge migration in the hot regions near the HV electrode plays a crucial role in creating the analogous ineffective region. The expansion of this analogous ineffective region results in a large voltage drop across the area near the ground electrode. Under these conditions, positive streamers can be triggered at the triple junction of the ground electrode.

Also, during the experiment we found that it is easier for the analogous ineffective region to expand when using multi-layer insulation structure than that of using single layer dielectrics. It is due to charge accumulation at the interface of multilayer structure. To reduce the expansion of the analogous ineffective region under an applied DC voltage at a temperature gradient, dielectrics with low thermal conductivity can be produced using bulk doping methods. However, these materials should only be produced while maintaining the mechanical and electrical properties of the original dielectrics, which makes the fabrication process a difficult task. Additionally, restraint of the electron migration in dielectric materials is also sensible. In this case, dielectric materials with deep traps may represent a good choice for their charge injection restraint properties. Admittedly, the concepts mentioned in this paper are simply promising candidates for restraint of the analogous ineffective region in dielectric materials. It should be noted that, during operation, the load of the energy transmission system suffers from daily variations that may result in temperature shifts in the HV conductors. During this process, the charge migration behaviour can be changed with respect to these temperature fluctuations and may lead to different charge distributions when compared with the distribution that occurs under stable temperature conditions. In addition, the corona that occurred at the triple junction of the ground electrode also introduces heat and increased numbers of charges to the bulk and the surface of the dielectrics, which increases the difficulty of charge migration investigations. Apart from these aspects, considerable work remains to be done as part of the fundamental research in this field.

## Method

### Experimental sample preparation

The experimental samples were epoxy resins blended with 99.9% pure α-Al_2_O_3_ micro-sized powders. The epoxy resins, the hardener and the powder were all purchased from Huntsman Advanced Materials and were mixed with a weight ratio of 100:38:330 under vacuum conditions before being poured into the mould. The curing stage was performed at 130 °C for 24 h in the insulation workshop of Shandong Taikai High Voltage Switch Co. Ltd. The samples with dimensions of 3 mm × 40 mm × 40 mm were prepared for the surface potential and surface flashover voltage measurement. The samples with dimensions of 0.4 mm × 40 mm × 40 mm were prepared for the space charge measurement.

### Surface potential detection system

**Figure 10:**
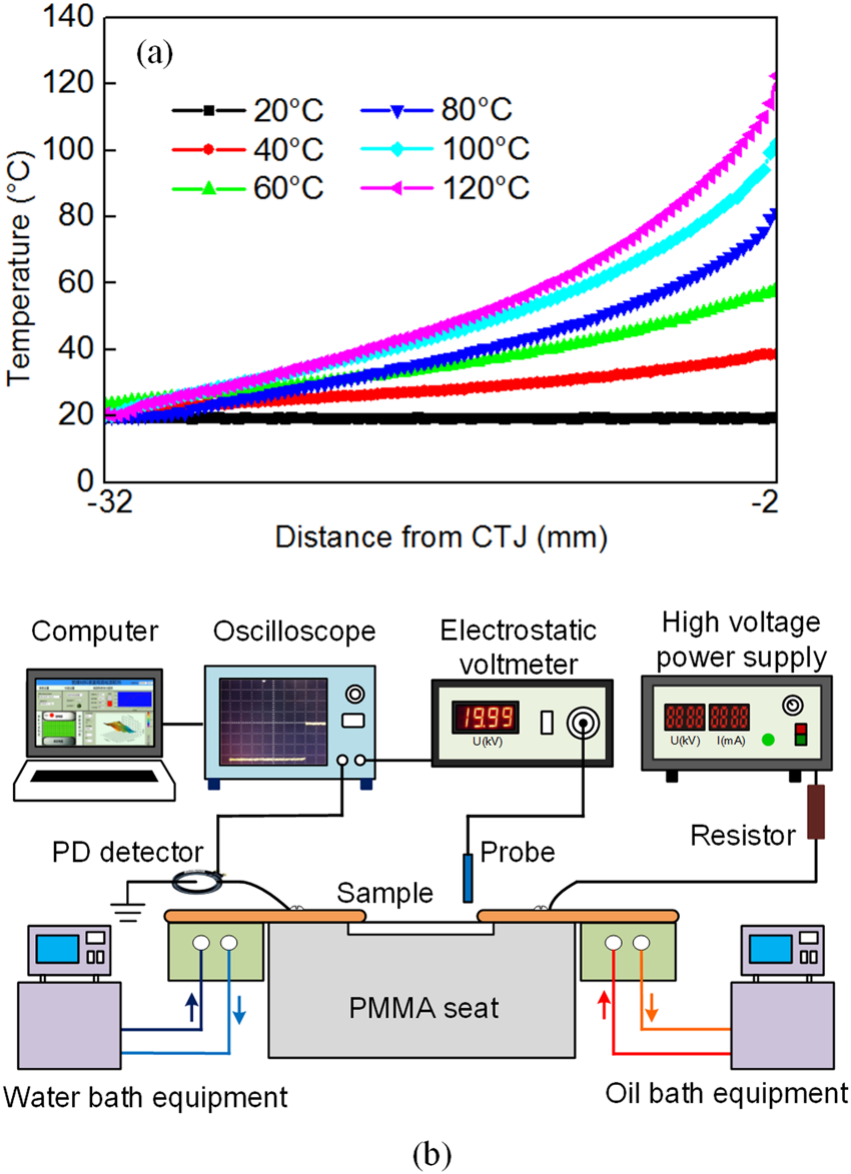
(**a**) The temperature gradient distribution under temperature from 20 °C to 120 °C and (**b**) the schematic illustration of the potential detecting system.

The experimental samples were prepared with dimensions of 3 mm × 40 mm × 40 mm and were pressed by two tapered rectangular alumina electrodes with dimensions of 3 mm × 34 mm × 64 mm that were placed in a sample seat made from poly(methyl methacrylate) (PMMA). Two cube heaters were fixed such that they adhered closely under the alumina electrodes on both sides of the sample seat, with one cube heater connected to the water circulation pump and the other connected to the oil circulation pump. During the experiments, to simulate the actual conditions, the HV electrode temperature is elevated from 20 °C to 120 °C in 20 °C increments, while the temperature of the ground electrode is controlled at 20 °C. A schematic diagram of the temperature gradient at each level is shown in Fig. 10(a). Each experiment was conducted 30 min after the temperature of the cube heater reached stability. The surface potential distribution is measured using a Kelvin-type probe (Trek 6000B-5C) linked to an electrostatic voltmeter (Trek 347). The distance between the probe and the sample surface is set at between 3 mm and 3.5 mm. The probe can shuttle from above the two electrodes within 4 s, where it is carried by a motion machine, and the surface potential data is recorded using an oscilloscope (LeCroy WaveRunner 610Zi). A voltage of −3 kV is applied and potential detection is carried out at times of 20 s, 120 s, 600 s, and 1200 s after the voltage is applied. A schematic illustration of the potential detection system is shown in Fig. 10(b).

### Surface flashover and corona detection

The surface flashover experiment was carried out in a dark room with a voltage boost rate of 50 V/s, while using the same PMMA seat that was used in the surface potential detection test. The experimental sample is cleaned with pure anhydrous ethanol for 60 min and is then baked for more than 6 h at 60 °C before the experiment. Ten samples were prepared for the tests under each temperature with the surface flashover voltage being recorded for each, and the average value is then calculated to be used as the final value. The pure anhydrous ethanol (99.9%) is used to clean the sample surface to remove any residual charge before the experiment begins. The corona was detected using a high frequency current transformer sensor (HFCT 100/50) with a wideband frequency detection range from 90 kHz to more than 20 MHz. The ambient temperature was 16 °C and the relative humidity was 18%.

### Thermal Characterization

The temperature distributions of the experimental samples were measured using an infrared thermal imager (Fluke Ti400). The temperature distributions was photoed after the temperature had reached stabled for 10 min.

### Space Charge Measurement

**Figure 11:**
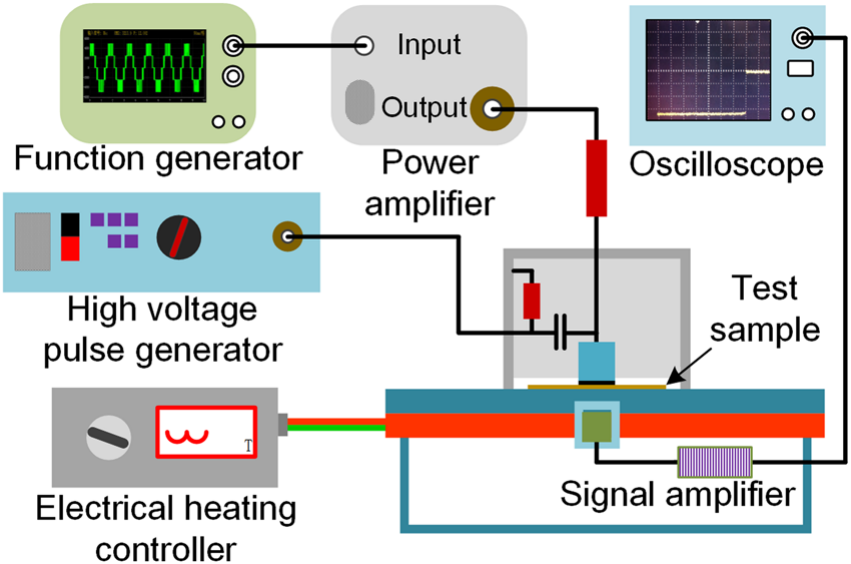
The schematic illustration of the PEA system.

The space charge measurements were carried out using the Pulsed Electroacoustic (PEA) measurement system. The signal is detected with a Lecroy WaveRunner 610Zi (1 GHz, 10 GS·s^−1^ digital oscilloscope). During the space charge test, a dc electrical field of 40 kV mm^−1^ and an electric pulse with an amplitude of 800 V and duration of 7 ns were applied to the experimental samples with a thickness of 400 um for 600 s. A piezoelectric transducer of polyvinylidene fluoride film with a thickness of 9 um was used to transform the acoustic signal into an electrical signal. Calibration for overshoot due to the nonlinear phase distortion was completed by the signal processing. In addition, the calibration for attenuation was finished to obtain the accurate information of the space charge profiles. The diagrammatic sketch of the PEA system is shown in Fig. 11.The charge injection amount in the samples can be calculated based on the charge density distribution according to

$$q(t)={\int }_{0}^{d}\rho (x,t)Sdx$$

where ρ is the charge density, *t* is the time, *x* is the spatial coordinate, *S* is the electrode area and *d* is the thickness of the sample^60^. The electrical field distribution can be obtained from the space charge distribution by using Poisson’s equation.
